# Investigation of the Proteolytic Functions of an Expanded Cercarial Elastase Gene Family in *Schistosoma mansoni*

**DOI:** 10.1371/journal.pntd.0001589

**Published:** 2012-04-03

**Authors:** Jessica R. Ingram, Salma B. Rafi, A. Alegra Eroy-Reveles, Manisha Ray, Laura Lambeth, Ivy Hsieh, Debbie Ruelas, K. C. Lim, Judy Sakanari, Charles S. Craik, Matthew P. Jacobson, James H. McKerrow

**Affiliations:** 1 Tetrad Graduate Program, University of California San Francisco, San Francisco, California, United States of America; 2 Department of Pharmaceutical Chemistry, University of California San Francisco, San Francisco, California, United States of America; 3 Department of Pathology, University of California San Francisco, San Francisco, California, United States of America; 4 Sandler Center for Drug Discovery, University of California San Francisco, San Francisco, California, United States of America; McGill University, Canada

## Abstract

**Background:**

Cercarial elastase is the major invasive larval protease in *Schistosoma mansoni*, a parasitic blood fluke, and is essential for host skin invasion. Genome sequence analysis reveals a greatly expanded family of cercarial elastase gene isoforms in *Schistosoma mansoni*. This expansion appears to be unique to *S. mansoni*, and it is unknown whether gene duplication has led to divergent protease function.

**Methods:**

Profiling of transcript and protein expression patterns reveals that cercarial elastase isoforms are similarly expressed throughout the *S. mansoni* life cycle. Computational modeling predicts key differences in the substrate-binding pockets of various cercarial elastase isoforms, suggesting a diversification of substrate preferences compared with the ancestral gene of the family. In addition, active site labeling of SmCE reveals that it is activated prior to exit of the parasite from its intermediate snail host.

**Conclusions:**

The expansion of the cercarial gene family in *S. mansoni* is likely to be an example of gene dosage. In addition to its critical role in human skin penetration, data presented here suggests a novel role for the protease in egress from the intermediate snail host. This study demonstrates how enzyme activity-based analysis complements genomic and proteomic studies, and is key in elucidating proteolytic function.

## Author Summary

Schistosome parasites are a major cause of disease in the developing world. The larval stage of the parasite transitions between an intermediate snail host and a definitive human host in a dramatic fashion, burrowing out of the snail and subsequently penetrating human skin. This process is facilitated by secreted proteases. In *Schistosoma mansoni*, cercarial elastase is the predominant secreted protease and essential for host skin invasion. Genomic analysis reveals a greatly expanded cercarial elastase gene family in *S. mansoni*. Despite sequence divergence, SmCE isoforms show similar expression profiles throughout the *S. mansoni* life cycle and have largely similar substrate specificities, suggesting that the majority of protease isoforms are functionally redundant and therefore their expansion is an example of gene dosage. However, activity-based profiling also indicates that a subset of SmCE isoforms are activated prior to the parasite's exit from its intermediate snail host, suggesting that the protease may also have a role in this process.

## Introduction

Schistosomes and related blood flukes are platyhelminth parasites of hosts ranging from fish to humans. In humans, schistosome infection leads to the disease schistosomiasis, which affects upwards of 200 million people worldwide [1]. Prevalent in developing nations, it is ranked second to malaria in terms of overall morbidity caused by parasitic disease [2], [3]. The parasite has a complex life cycle, infecting both an intermediate snail host and a definitive human host. At multiple life cycle stages, the parasite must migrate through host tissue, and breach substantial structural barriers, including the extracellular matrix [4]. The process of early human infection is well-characterized: the multi-cellular larval stage, termed cercaria(e), directly penetrates host skin in a process facilitated by secretions from a glandular network that runs the length of the larval body. These secretions contain multiple histolytic proteases [5], [6]. In *Schistosoma mansoni*, the most of abundant of these is cercarial elastase (SmCE), an S1A serine protease named for its ability to digest insoluble elastin.

Completion of the *S. mansoni* genome sequence revealed an expanded cercarial elastase gene family, including eight full-length genes. Based on sequence identity, these isoforms were classified as belonging to one of two groups: Group I, comprised of SmCE1a.1, SmCE1a.2, SmCE1b and SmCE1c; and Group 2, comprised of SmCE 2a.1, SmCE2a.2, SmCE2a.3 and SmCE2b. Corresponding proteins were identified for SmCE 1a, 1b and 2a. SmCE 1c was determined to be a pseudogene, and no corresponding protein has been identified for SmCE2b transcript. Multiple CE isoforms exist in the related species *Schistosoma haematobium*, including homologs of SmCE1a, 1b, 2a and 2b isoform types [7], [8] The expansion of this family appears to be limited to the human-specific schistosomes: *Schistosoma japonicum* encodes only a single cercarial elastase gene, and there is substantial evidence that this species, along with the avian-specific schistosomes, utilizes another class of protease in host invasion [7], [9]–[12].

The expansion of the cercarial elastase gene family in *S. mansoni*, and the sequence divergence among isoforms, suggests two alternative evolutionary scenarios: that gene duplication has allowed for differential regulation or diversified substrate specificity, in order to facilitate multiple functions in host invasion; or that SmCE gene duplication directly enhances fitness by simply increasing the amount of protease present. In the absence of robust genetic tools for studying schistosome biology, we decided to take a biochemical approach to study the functions of SmCE isoforms, using a combination of quantitative PCR, activity-based profiling and computer modeling to identify unique characteristics of the isoforms. Here, we present data that supports the hypothesis that expansion of the SmCE gene family in *S. mansoni* directly increases gene dosage, *i.e.* the number of copies of SmCE genes in the *S. mansoni* genome, which is likely to lead to a corresponding increase in SmCE protein levels. Moreover, analysis of SmCE isoform expression and activity throughout the parasite life cycle confirms that the protease is present and active very early in cercarial development, which suggests a potential role for the protease in egress from the intermediate snail host.

## Methods

### Ethics Statement

All vertebrate animal studies were performed in accordance with the USDA Animal Welfare Act and the Guide for the Care and Use of Laboratory Animals of the National Institutes of Health. The protocol was approved by the Institutional Animal Care and Use Committee of the University of California, San Francisco (Approval #086607-01).

### Cataloging of cercarial elastase genes in *Schistosome* species

Cercarial elastase genes for *S. mansoni*, *S. haematobium* and *S. japonicum* were identified by searching all three annotated genomes in the SchistoDB website (http://beta.schistodb.net) using the Gene Ontology (GO) Terms “endopeptidase” and “cercarial protease” or by BLAST analysis using the SmCE1b protein sequence as a query.

### Collection of parasite material

Parasite material originates from a Puerto Rican isolate of *S. mansoni* maintained by BEI Resources (Rockville, MD), and passaged through the intermediate snail host, *Biomphalaria glabrata*, and the Golden Syrian hamster *Mesocricetus auratus* (Simonsen Labs) or BALB/C mice (Jackson Labs, Bar Harbor, ME) at the University of California, San Francisco. Animals were maintained and experiments carried out in accordance with protocols approved by the Institutional Animal Care and Use Committee (IACUC) at UCSF. Adult worms were harvested as previously described [13]. Eggs were collected from infected livers harvested from euthanized hamsters. Briefly, livers were mechanically disrupted in a Waring blender on a low setting in 1× PBS/ trypsin. The resulting lysate was incubated at 37°C for 2 hrs, rinsed with 1× PBS, and liver material was allowed to sediment. Sedimented material was then transferred to a Petri dish, swirled, and eggs were collected from the center of the dish with a transfer pipette. Miracidia were collected by inducing egg hatching with light exposure for 30 min in distilled water. Miracidia that swam towards the light source were collected with a transfer pipette. Miracidia were then used to infect *Biomphalaria glabrata* snails *en masse*. Roughly 100 snails, just under 1 cm each, were incubated with several thousand miracidia overnight. Individual snails were then analyzed for signs of infection. Forty days *p.i.*, whole hepatopancreases were dissected from infected snails. Cercariae were harvested using a previously described method [14]. Collection of lung stage schistosomules was initiated by subcutaneous infection of 6-week-old BALB/C mice with several hundred cercariae. One week *p.i.*, mice were euthanized and schistosomules were harvested by perfusion.

### RNA and protein isolation

All parasite material was stored at −80°C; a portion of each sample was stored in Trizol (Life Tech) for subsequent RNA extraction. Total RNA was isolated from frozen tissue by homogenization and incubation in Trizol at 65°C for 5 min. Total nucleic acid was subsequently collected by phenol/chloroform extraction. The aqueous phase was then loaded onto a Stratagene RNA purification column (Stratagene) and purified following the manufacturer's protocol, with the inclusion of an on-column DNase treatment step. First-strand cDNA was then generated from this material using a first-strand synthesis kit and oligo d(T) primer (Invitrogen). Remaining frozen samples were lysed in 100 mM Tris, pH 8 and 0.1% NP40, briefly sonicated to disrupt tissue, and spun down for 15 min at 16,000 rcf at 4°C; the resulting supernatants were saved as the soluble protein lysates.

### Quantitative RT-PCR analysis

The following TaqMan primer/probe sets, containing a 5′ fluorescein amidite (FAM) / 3′ non-fluorescent quencher (NFQ) as the reporter/quencher pair on each probe (LifeTech, Carlsbad, CA) were designed to distinguish between SmCE1(a and b), SmCE2a and SmCE2b mRNA sequences: SmCE1 Forward 5′-TTA AGG TGG CAC CAG GAT ATA TGC-3′; SmCE1 Reverse 5′-TGA GTG TCT GTG CGA TTG GT-3′; SmCE1 Probe 5′-FAM-TCG TGC CGA CAT ACA AG-NFQ-3′; SmCE2a Forward 5′-CCA CCA CTG GGA ATC CTA TTT GT-3′; SmCE2a Reverse 5′-CCT GGT GCG GTG ATT TGC-3′; SmCE2a Probe 5′-FAM-AAG CGG CGT ATG TGT TC-NFQ-3′; SmCE2b Forward 5′-GGC ATG CAG ACA CAA ACG T-3′; SmCE2b Reverse 5′-TGT CAC CTG GAC CAG CTA TTT G-3′; SmCE2b Probe 5′-FAM-CAG GTC CGA ACT CAA AG-NFQ-3′. In addition, the following primers/probe were designed against SmGAPDH as a reference gene: SmGAPDH Forward 5′-ACT CAT TTA CGG CTA CAC AAA AGG T-3′; SmGAPDH Reverse 5′-CAG TGG AAG CTG GAA TAA TAT TTT GCA-3′; SmGAPDH Probe 5′-FAM-CTC GCC ATA ATT TTG-NFQ-3′. Reactions were performed in 10 µL 2× ABI Gene Expression Mix, 1 µL Pre-mixed Primer/Probe, 7 µL dH_2_O and 1 µL cDNA template. Cycle conditions were as follows: 95°C for 10 min; 40 cycles of 95°C for 15 s, 60°C for 1 min. Reactions were run on an MX3005P quantitative thermocycler (Stratagene).

### Western blot analysis

Bradford assays were performed to quantify total protein amount and an equal protein concentration of lysate from each life cycle stage was added to 4× reduced SDS-PAGE loading dye (LifeTech). A total volume of 15 µL was loaded onto a 10% Bis-Tris SDS-PAGE gel (LifeTech). Bands were then transferred by electroblotting to a PVDF membrane, and visualized by Ponceau staining to check for equal transfer of all samples (Figure S1). Blots were incubated with a 1∶1000 dilution of polyclonal SmCE [14], followed by a 1∶5000 dilution of anti-rabbit IgG-HRP (GE Healthcare) and 2 ml ECL reagent (GE Healthcare). Blots were exposed to Kodak High Sensitivity BioFilm for visualization (Kodak, Rochester, NY).

### Activity-based probe labeling

Bradford assays were performed to quantify total protein amount, and an equal protein concentration of lysate from each life cycle stage was used. For each sample, 50 µL lysate was added to 100 µL 50 mM Tris, pH 8, 150 mM NaCl. This mixture was then pre-cleared by incubating for 5 min with 50 µL avidin beads (Pierce Biotechnology, Rockford, IL) pre-equilibrated in 50 mM Tris, pH 8. The beads were pelleted by centrifugation for 30 sec at 3000 rcf, and the soluble portion was moved to a new microfuge tube. The probe biotin-nVPL-O(Ph)_2_ (biotin-PEG-norleucyl-valyl-prolyl-leucyl-diphenyl phosphonate) was synthesized following a previously described method, added for a final concentration of 5 µM, and incubated for 1 hour at room temperature [15]. To stop the reaction, 400 µL 1 M guanidine-HCl was added and the entire reaction was run over a 5 kDa Amicon filter (Millipore, Bedford, MA). The filter was washed in 400 µL 1 M guanidine-HCl, 400 µL 50 mM Tris, pH 8 and concentrated to a final volume of 30 µL. Ten microliters were then added to 6 µL SDS-PAGE reducing dye and loaded onto a 10% Bis-Tris SDS-PAGE gel (LifeTech). Bands were then transferred by electroblotting to a PVDF membrane, and visualized by Ponceau staining to check for equal transfer of all samples (Figure S2). Blots were incubated with a 1∶2000 dilution of Avidin-HRP (Pierce) and 2 mL ECL reagent (GE Healthcare). Blots were then exposed to Kodak High Sensitivity BioFilm for visualization (Kodak, Rochester, NY).

### Snail hepatopancreas section preparation and staining

Hepatopancreases containing mature schistosome daughter sporocysts were dissected from infected snails and placed in 4% paraformaldehyde in phosphate buffer pH 7.4. Following an overnight fixation at 4°C, the tissue was washed in 0.1 M phosphate buffer with 3% sucrose, dehydrated in graded acetones, and then infiltrated with glycol methacrylate monomer. The tissue was then transferred to molds containing the complete embedding mixture, and placed under vacuum for 12 hours. The entire procedure was carried out at 4°C. The hardened blocks were then sectioned at 2 µm and air-dried. To identify active protease in developing cercariae, the esterase procedures of Li *et al* for alpha-naphthylacetate (with and without sodium fluoride) and naphthol AS-D chloroacetate were performed at pH 8 [16]. To definitively identify activity as due to the serine protease of cercariae, two chloromethyl ketone inhibitors were employed. The first is a known inhibitor of the protease, Z-AAPF-CMK (alyl-alyl-prolyl-phenyl-chloromethyl ketone), and the second is known not to inhibit the protease, Z-FPR-CMK (phenyl-prolyl-argyl-chloromethyl ketone) [14]. Sections were pre-incubated with inhibitors for one hour prior to initiation of the esterase reaction. Cleaved substrate gives a distinctive color reaction (orange/red) visualized by light microscopy.

### Computational modeling

We used the structure of bovine alpha-chymotrypsin refined at 1.68 Å resolution (pdb ID: 4CHA) as a template to construct models for the 8 isoforms SmCE1a.1, SmCE1a.2, SmCE1b, SmCE1c, SmCE2a.1, SmCE2a.2, SmCE2a.3 and SmCE2b. Only chains B and C of chymotrypsin were used. The percent identities for each one with the chymotrypsin structure (chains B and C of 4CHA) are 23.7%, 23.3%, 22.6%, 20.6%, 23.7%, 23.7%, 23.7% and 21%, respectively. The models were built by using the Prime software (version, 2.2, Schrödinger LLC, New York, NY, 2010). Secondary structure prediction was performed. The sequence alignment generated by Prime was manually modified to allow for the regions of insertions or deletions in the target to fall in the loop regions. The models generated were aligned to the template and the backbone RMSD values calculated to validate the models using Prime software protein alignment tool. The backbone RMSD values are 0.83 Å, 0.72 Å, 0.8 Å, 0.76 Å, 0.72 Å, 0.72 Å, 0.72 Å and 0.64 Å, respectively. The Protein Structure Validation Software Suite (PSVS server) was used to validate our models [17]. These values are provided as Table S1. The sequence alignment was generated using ClustalW [18].

## Results

### All SmCE isoforms are expressed primarily in the intramolluscan and larval stages

**Figure 1:**
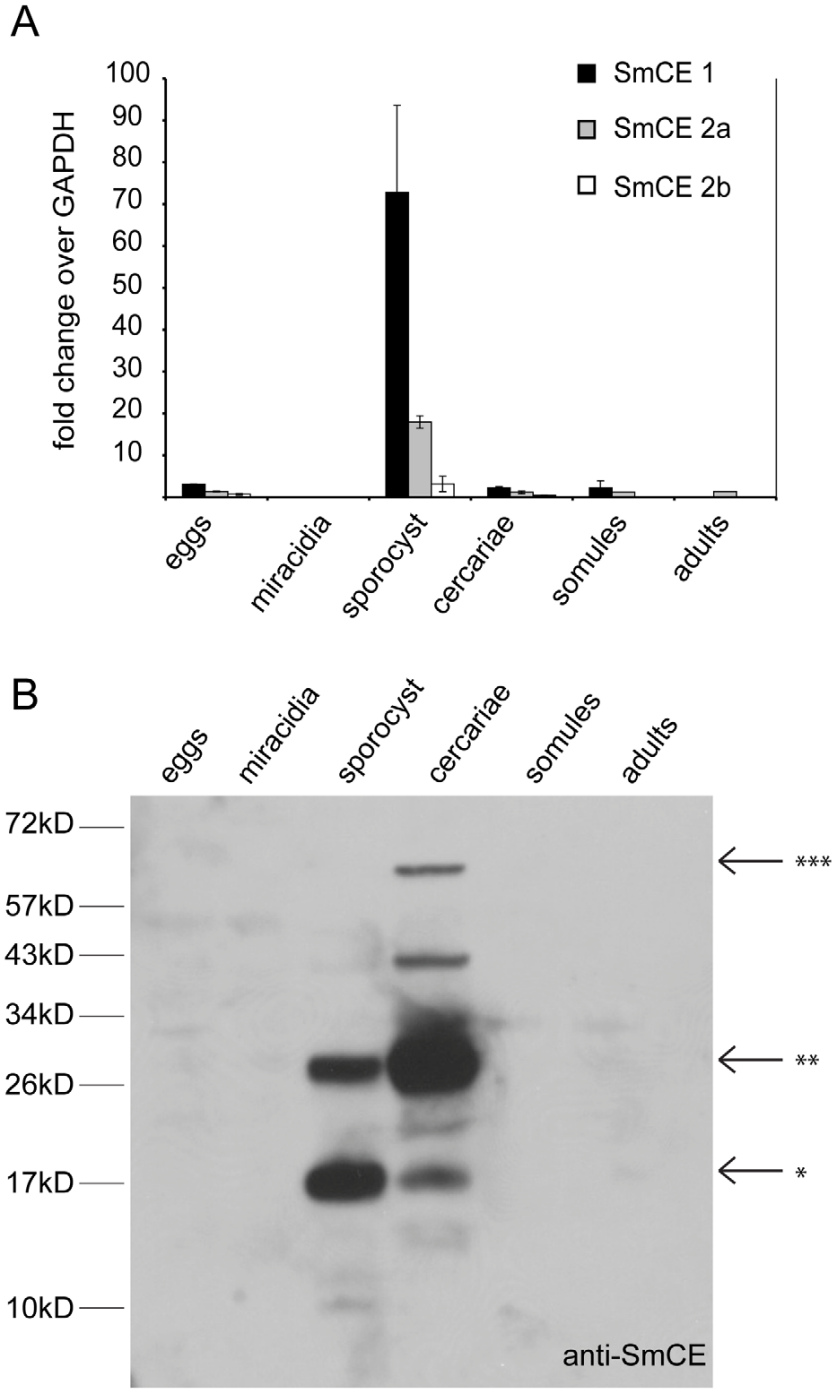
All SmCE isoforms show similar mRNA and protein expression patterns throughout the *S. mansoni* life cycle. (A) SmCE1(a and b), SmCE 2a and SmCE2b mRNA levels were monitored by quantitative RT-PCR. (B) Protein levels of SmCE were monitored in lysates from all life cycle stages by immunoblot with a polyclonal antibody raised against recombinant SmCE. A 26 kD band (**) correlates to the full-length SmCE protein, whereas a 17 kD band (*) correlates to a previously identified auto-degradation product and a 68 kD band (***) correlates to a complex of SmCE and its endogenous serpin, SmSerpQ [14], [20]. An additional band at approximately 43 kD is likely a dimerized SmCE as suggested by size exclusion chromatography showing activity at this molecular weight [33].

Annotation of the *S. mansoni* genome revealed a total of eight full-length SmCE isoforms; based on amino acid sequence homology these can be grouped, as can previously identified isoforms, into two major classes [7]. To determine if all SmCE isoforms were expressed at the same parasite life cycle stages, we purified total RNA from all major stages. We then performed quantitative RT-PCR with TaqMan primer/probe combinations that were specific to the different isoform subsets (Figure 1A). SmCE1a and b are highly similar at the nucleotide sequence level, so a primer/probe set common to both was made, in addition to sets specific for SmCE2a and SmCE2b. Transcript levels were normalized to SmGAPDH, which is stably expressed throughout the entire *S. mansoni* life cycle (Figure S3). SmCE1(a and b), SmCE2a and SmCE2b mRNA expression is confined to the daughter sporocyst and cercarial stages, with significantly more transcript present in the thirty day old daughter sporocysts, which develop in the hepatopancreas of the intermediate snail host. The relative levels of isoform mRNA expression correlate with the number of genes corresponding to each isoform: SmCE1(a and b) are encoded by the most genes, and are the most abundant SmCE transcripts. SmCE2b, for which no protein has been identified, comprises the smallest portion of SmCE transcript.

Next, we sought to determine the protein expression profile of SmCE. Soluble protein was extracted from all parasite life cycle stages, and SmCE protein was detected by immunoblot with polyclonal antiserum raised against recombinant, denatured SmCE1a. Given that the antiserum was raised against whole protein and that there is a high level of similarity between isoforms, it is likely that the antibody cross-reacts with a number of SmCE isoforms [7] (Figure 1B). While not isoform specific, this antibody revealed that the majority of SmCE protein is expressed in the late sporocyst (40 *d.p.i*) and cercarial stages, but is absent in the juvenile, or lung-stage, parasites one week after initial infection. The antibody does not cross-react with uninfected snail hepatopancreas (Figure S4). This correlates with previous studies that suggest SmCE is produced very early in cercarial development within the daughter sporocyst and that the contents of the acetabular glands are rapidly exhausted during the course of skin invasion [19].

### SmCE is activated prior to exit from the intermediate snail host

**Figure 2:**
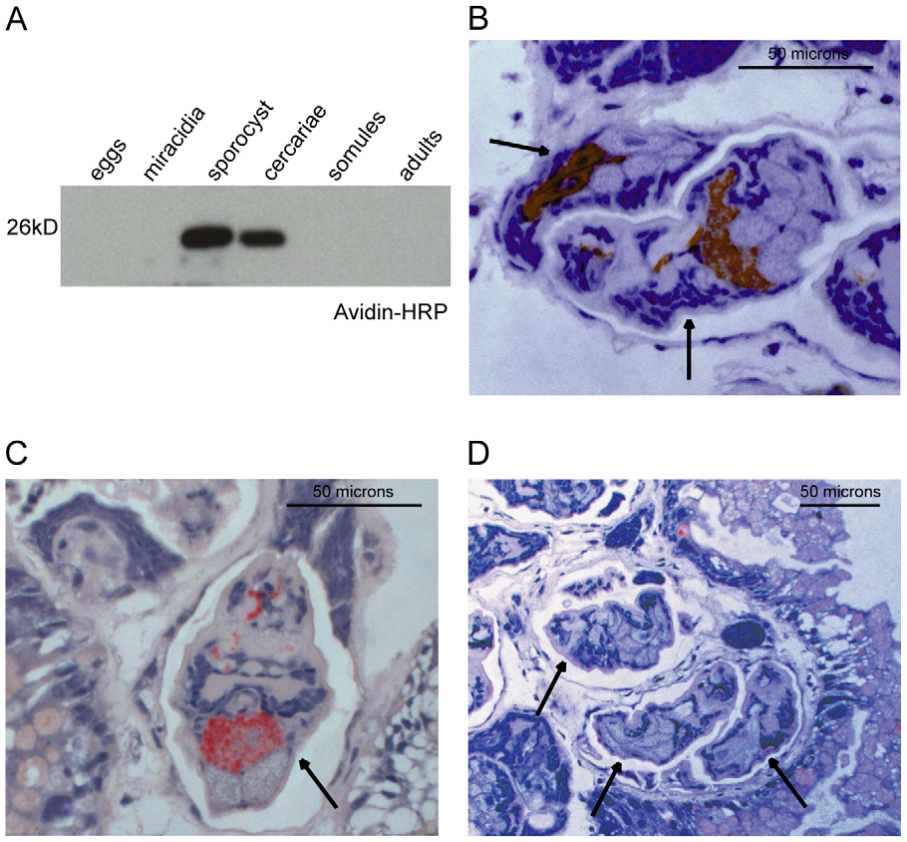
SmCE is active within the intermediate snail host. (A) Avidin-HRP blot of lysates made from various *S. mansoni* life cycle stages incubated with biotin-nVLP-(OPh)_2_. No additional bands were visible on the blot after a three-minute exposure with a highly sensitive ECL reagent. (B) Histological sections of a patent *B. glabrata* hepatopancreas that contained developing cercariae were treated with a general serine esterase stain (red staining) to detect serine protease activity. Similar sections were pre-treated with either (C) a non-CE specific serine protease inhibitor, FPR-CMK, or (D) a CE-specific inhibitor, AAPF-CMK, prior to esterase staining. Arrows indicate the developing cercariae within the snail tissue. Immunoperoxidase localization of SmCE using an antibody raised against SmCE has been published, and esterase staining shown here localizes to similar regions of the cercariae, namely the preacetabular glands and their ducts [21].

Given the frequent presence of endogenous protease inhibitors and activators, protein levels alone are not always indicative of proteolytic activity, and tracking activity is critical to understanding the function of a given protease. SmCE is expressed as a zymogen and contains a short prodomain that must be removed in order for the protein to become active; moreover, SmSerpinQ, a serpin (serine protease inhibitor) with specificity against cercarial elastase provides additional post-translational regulation of protease activity [14], [20]. We therefore sought to specifically identify where active SmCE is present in the *S. mansoni* life cycle. To this end, we made use of a biotinylated phosphonate probe, biotin-nVPL-O(Ph)_2_, that had previously been shown to bind to active SmCE [9]. The presence of phenoxy groups on this molecule increases the electrophilicity of the phosphorus atom, promoting nucleophilic attack of this atom by the active site serine of SmCE. This in turn leads to a covalent linkage. In this way, only active protease (*i.e.*, protease without a prodomain, or in complex with inhibitor) is biotinylated. Lysates from all major life cycle stages were incubated with the inhibitor for an hour at room temperature, run on an SDS-PAGE gel and transferred to PVDF membrane for detection with HRP-conjugated avidin. This labeling revealed that active SmCE was present in both six-week daughter sporocysts and shed cercariae (Figure 2A).

While SmCE is known to be active in the invasive cercariae, where it facilitates transit through host skin, the finding that it was activated prior to exit from the intermediate snail host was surprising. Given the possibility that a non-physiological activating factor was present in the lysate, we further explored the activation of the enzyme in histological cross-sections of snail hepatopancreas tissue. Snails were sectioned approximately six weeks after infection, and sections were preserved in paraformaldehyde to minimize protein cross-linking and thereby preserve enzyme function. Treatment with a general serine esterase stain led to distinct visual staining in both the acetabular cell bodies and the ducts leading from acetabular cells to the anterior end of developing larvae (Figure 2B). These morphological features are prominent and easily identifiable in the developing cercariae, which is itself contained within the daughter sporocyst [21]. Pre-treatment with Z-AAPF-CMK, a known inhibitor of SmCE, abolished stain reactivity (Figure 1C), whereas treatment with Z-FPR-CMK, to which the SmCE is largely insensitive, had no effect on esterase staining (Figure 1D) [22]. This led to the conclusion that visualized staining was specific to SmCE activity within the developing cercariae.

### Homology modeling predicts key differences in the SmCE isoforms substrate-binding sites

**Figure 3:**
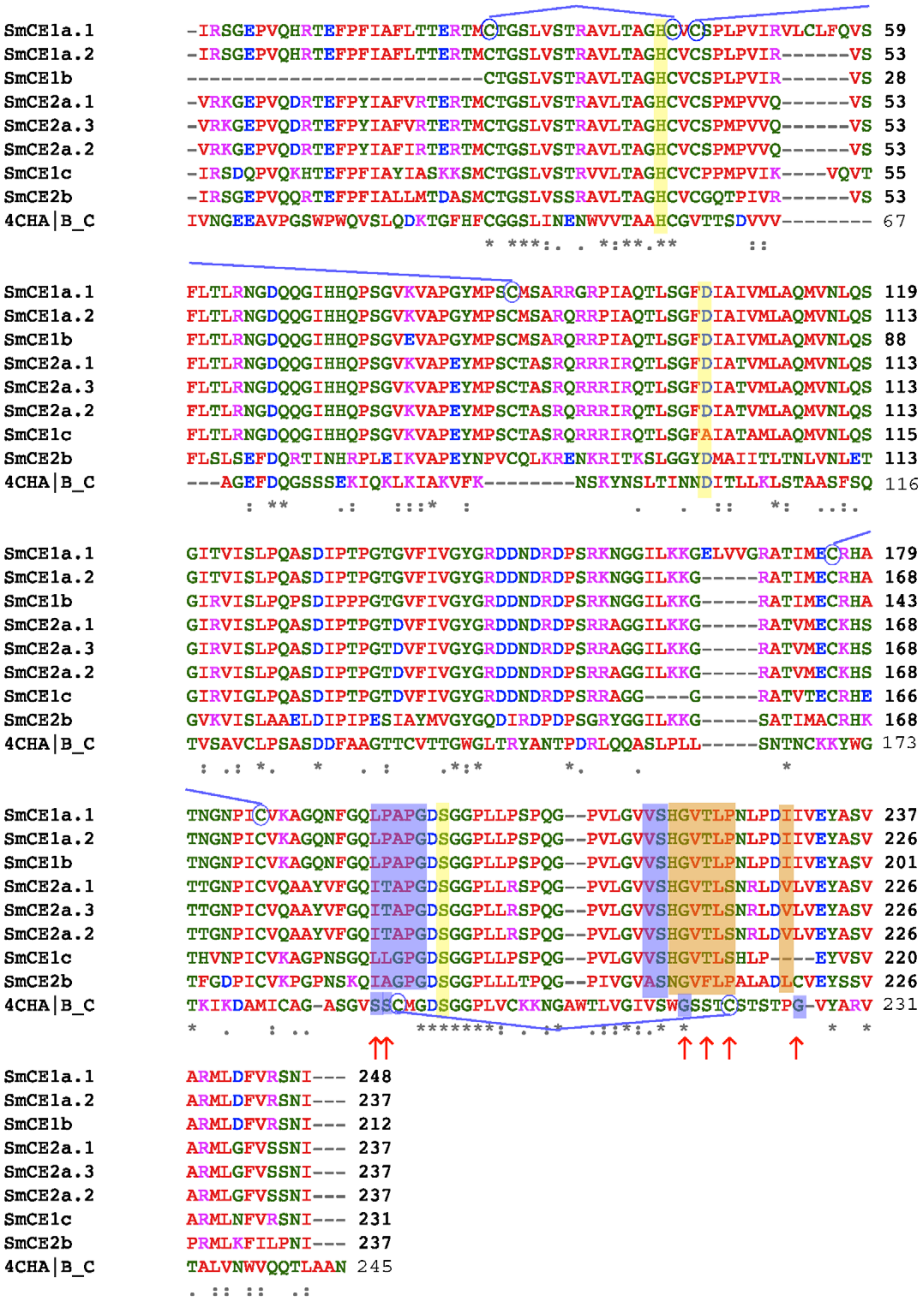
Protein sequence alignment reveals that SmCE isoforms can be classified as belonging to one of two main groups. The amino acid sequences of mature SmCE isoforms amino acid sequences and bovine chymotrypsins (PDB ID: 4CHA) B and C chains were aligned using ClustalW. Active site residues are highlighted in yellow. Cysteines forming disulfide bridges are circled. Residues predicted to comprise the S1 binding pocket are shaded blue; residues predicted to comprise the S4 binding pocket are shaded orange. Residues depicted in the binding site models (Figure 4) are indicated with red arrows.

In order to further explore whether SmCE gene expansion has led to diversified function of protease isoforms, we next looked at the amino acid sequence variation between isoforms. Computational models of all eight full-length SmCE isoforms were generated by alignment to bovine chymotrypsin (pdb ID: 4CHA) (Figure 3). Models of SmCE1a.2, SmCE2a.2 and SmCE2b, as representative members of each isoform group, are presented here. All amino acid positions are described using canonical chymotrypsin numbering. While SmCE is predicted to have the basic α/ß fold common to all chymotrypsin-like serine proteases, there are several notable structural features that are largely unique to SmCE, as has been previously noted [22]. These include a missing disulfide bond that is formed between Cys116 and Cys220 in chymotrypsin, and that defines the S3 binding pocket. In this regard SmCE resembles the structure of rat mast cell protease II [23].

**Figure 4:**
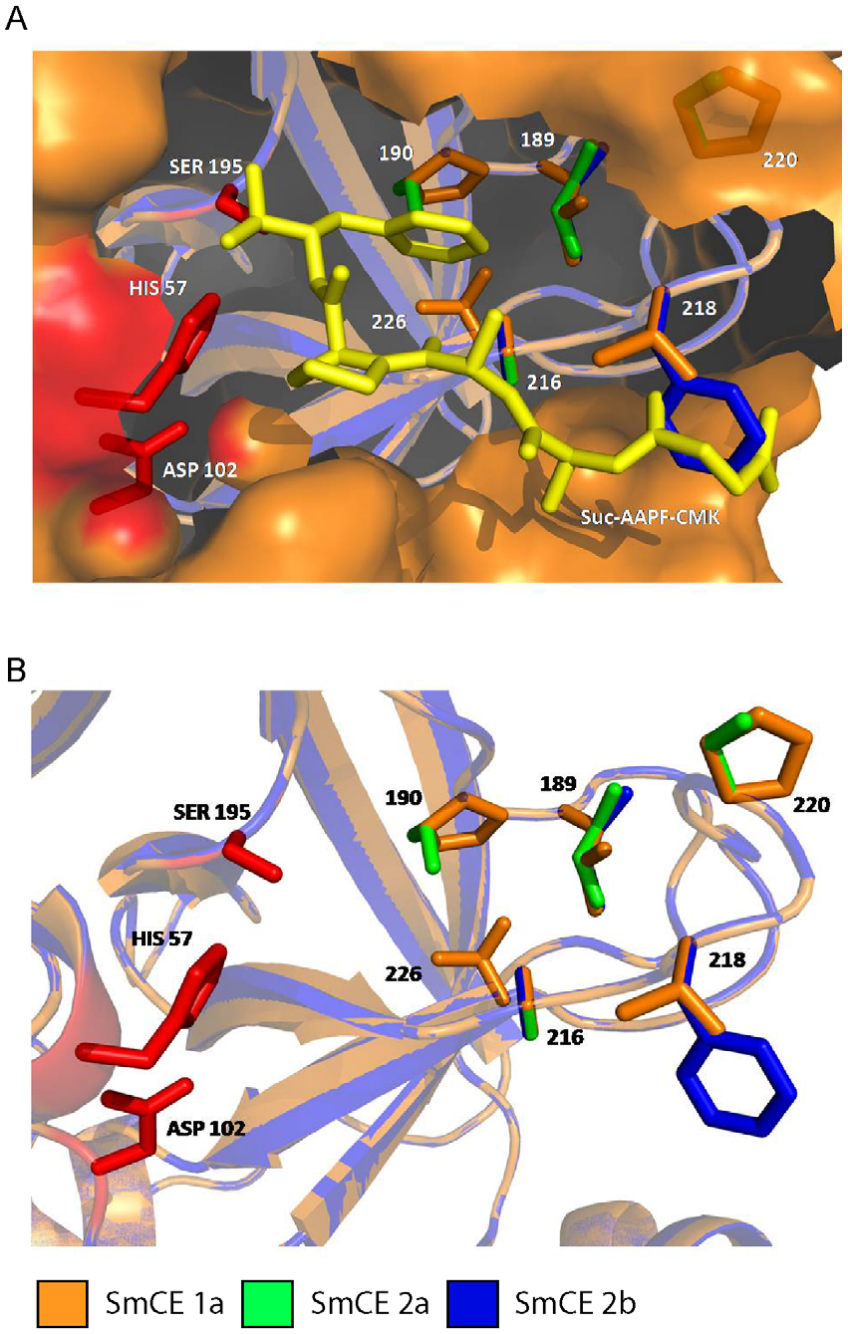
Computational modeling predicts key differences in the substrate-binding pocket of among SmCE isoforms. SmCE 1a.1, SmCE 2a.1 and SmCE2b were chosen as representative members of each isoform group and modeled on bovine chymotrypsin (PDB ID: 4CHA). Overlaid models of the substrate binding pocket are presented here with both (A) AAPF-CMK (yellow) docked in the active site and (B) the active site without substrate. Residues predicted to be key binding site determinants are presented as ball-and-stick models, and colored according to isoform group. Active site residues are depicted in red. All amino acid positions are presented according to canonical chymotrypsin numbering.

While our models predict that amino acid variations between isoforms are scattered throughout the surface of the protein, several notable structural differences within the substrate-binding pocket are predicted to exist among SmCE isoform groups (Table 1, Figures 4 and S5). In protease nomenclature, the position where the amino acid N-terminal to the scissile bond of the substrate binds the active site of the protease is referred to as the S1 pocket, and the next site is the S2, etc. The corresponding C-terminal positions are the S1′, S2′, etc. In chymotrypsin, Ser189 and Ser190 form the base of the S1 pocket [24]; in all SmCE isoforms, a hydrophobic leucine or isoleucine is predicted to occupy the 189 position (Figure 3; Figure 4a). Position 190 is more varied, with a threonine in SmCE2a, an alanine in SmCE2b and a proline in SmCE1a and 1b. Moreover, while Gly216 is conserved between trypsin, chymotrypsin and SmCE isoforms, position 226 is a leucine rather than a glycine, suggesting that that architecture of the S1 pocket is distinctly hydrophobic in most SmCE isoforms, which is consistent with previous biochemical studies of substrate specificity [25].

**Table 1:** Key amino acid positions that form the S4 and S1 pockets in SmCE isoforms.

	S4 Pocket			S1 Pocket			
Amino Acid Position	217	218	220	189	190	216	226
**SmCE Isoform**							
**SmCE1a.1**	Val	Thr	Pro	Leu	Pro	Gly	Leu
**SmCE1a.2**	Val	Thr	Pro	Leu	Pro	Gly	Leu
**SmCE1b**	Val	Thr	Pro	Leu	Pro	Gly	Leu
**SmCE1c**	Val	Thr	Ser	Leu	Leu	Gly	N/A
**SmCE2a.1**	Val	Thr	Ser	Ile	Thr	Gly	Ile
**SmCE2a.2**	Val	Thr	Ser	Ile	Thr	Gly	Ile
**SmCE2a.3**	Val	Thr	Ser	Ile	Thr	Gly	Ile
**SmCE2b**	Val	Phe	Pro	Ile	Ala	Gly	Cys
**Bovine Chymotrypsin**	**Ser**	**Ser**	**Cys**	**Ser**	**Ser**	**Gly**	**Gly**

Bovine chymotrypsin is provided as a reference.

The S4 pocket varies significantly between SmCE2b and the remaining SmCE isoforms (Figure 4). SmCE2b is unique in having a phenylalanine rather than a threonine predicted to occupy position 218, again increasing hydrophobicity in this pocket and also potentially leading to a steric clash with substrates or inhibitors with a large hydrophobic side chain at this position (Figure 4a). The charges of the prime side substrate-binding pocket also vary significantly between SmCE2b and other SmCE isoforms. The consequences of this are of ongoing investigation (*O'Donoghue et al, manuscript in preparation*), but suggest that SmCE2b may have a somewhat divergent substrate binding preference compared to other SmCE isoforms.

## Discussion

Gene duplication is abundant in all sequenced eukaryotic genomes [26]. Parasites frequently regulate gene expression through gene duplication. This is especially true for protozoan parasites, like *Leishmania* and *Trypansoma spp*, which contain tandem arrays of duplicated genes throughout their respective genomes [27], [28]. This presumably compensates for a lack of transcriptional regulatory control (both species transcribe genes as polycistronic mRNAs), with greater gene number resulting in greater gene expression. In other parasite species, along with most eukaryotes, gene duplication frequently results in either an altered or novel gene function, and there are several examples of this that specifically involve protease gene families, including the duplication and functional divergence of the cathepsin L family in the liver fluke *Fasciola hepatica*, and that of several aspartic proteases in the nematode *Strongyloides ratti* [29], [30].

The expansion of the cercarial elastase gene family by gene duplication in *S. mansoni* appears to involve both an increase in gene dosage for a critical enzyme during a crucial life cycle stage transition—host skin invasion–and may also represent the emergence of a new function of a serine protease as an invasive enzyme. The tight temporal regulation of the expression and activity of all SmCE isoforms throughout the parasite life cycle suggests that they share a conserved function. Moreover, the residues of the active site and the surrounding residues that determine the substrate specificity are largely conserved among the isoforms, suggesting that they share a common pool of substrates, most likely those present in human skin [12]. Slight variations in the substrate binding pockets of individual isoforms, however, may expand the range of available substrates in skin.

A notable exception to this is the SmCE 2b isoform. A homolog of this isoform is present in *S. japonicum* and is likely to be the ancestral gene of this family [10]. Despite the presence of transcript in the developing cercariae, no protein corresponding to this isoform has been identified in *S. mansoni*, or *S. japonicum*, although the latter case is a point of ongoing debate [5]–[10]. However, the fact that SmCE 2b is actively transcribed and that the catalytic triad has been preserved by evolution suggest that the protein is functional. In addition, homology modeling predicts a greater level of divergence in the substrate-binding pocket of SmCE 2b compared to the other isoforms, notably in the S4 pocket, suggesting that it may have a different substrate preference than its cohorts.

The zoonotic *S. japonicum*, the avian-specific schistosome species and the more distantly related liver flukes are reported to use papain-like cysteine protease, rather than a serine protease, to facilitate initial invasion of their respective hosts [10], [11], [29]. Based on current genome data, the expansion of the cercarial elastase gene family coincides with increased selectivity of humans as the definitive host of *S. mansoni*. A plausible model is that the initial duplication of the ancestral gene allowed for mutations within the substrate-binding site of the duplicated gene to enhance activity against substrates present in human skin, including immune response proteins [12], [31]. Subsequent gene duplication events in the *S. mansoni*/*S. haematobium* lineage then increased gene expression of the human substrate-specific isoforms that comprise the Group 1 / Group 2a SmCE gene families. This model is further supported by the recent sequencing and publication of the *S. haematobium* genome, which identifies 6 cercarial elastase genes in this species [8]. Only two of these annotated genes contain the full-catalytic core of the protease: Sha_300711, with 84% amino acid identity to SmCE2b and 69% amino acid identity to SjCE, and Sha_300049, with 72% amino acid identity to SmCE1a.1 (Figure S6). Further curation of the *S. haematobium* genome will allow for identification of additional full-length CE sequences, but the presence of both type CE1 and CE2b isoforms implies that amplification of the cercarial elastase gene family from the ancestral 2b gene is likely to have occurred prior to the divergence of the *S. haematobium* and *S. mansoni* lineages.

This model presupposes that SmCE 2b functions differently in the skin invasion process, or functions elsewhere in the parasite life cycle. It is possible the SmCE2b has a greater role in egress from the intermediate snail host, since data presented here suggests that at least a subset of SmCE isoforms are activated prior to the parasite exiting the intermediate host. While a previous microarray study by Gobert *et al* suggest the unique up-regulation of SmCE2b in 5 day cultured schistosomules, only trace amounts of SmCE2b transcript were found in the *in vivo* lung schistosomules in this study [32]. Future work, including the development of more sensitive molecular probes to track individual isoform activity and recombinant expression of all SmCE protease isoforms, are sure to lead to further elucidation of the functions of this gene family.

### Accession Numbers

SmCE1a.1 (GenBank: AAC46967.1); SmCE1a.2 (GenBank: CAY19049.1 or *S.m.* genome: Smp_119130); SmCE1b (*S.m.* genome: Smp_115980); SmCE2a.1 (GenBank: AAM43941.1); SmCE1c (GenBank: AAC46968.1); SmCE2a.2 (GenBank: CAZ28356.1 or *S.m.* genome Smp_006510); SmCE2a.3 (GenBank: CAY18611.1 or *S.m.* genome: Smp_112090); SmCE2b (GenBank: AAM43942.1 or *S.m.* genome: Smp_006520).

## Supporting Information

Table S1 **Z-scores of models.** The Protein Structure Validation Software Suite (PSVS server) was used to validate our models. There is no clear threshold score to define ‘good’ and ‘bad’ structures. However, since 2006 the NorthEast Structural Genomics Consortium has required that all NMR and X-ray crystal structures have Z scores>−5.
(TIF)

Figure S1 **Ponceau stain of anti-SmCE immunoblot.** A-eggs; B- miracidia; C-daughter sporocyst; D-cercariae; E-lung stage somules; F-lung control; G-adult worms.
(TIF)

Figure S2 **Ponceau stain of avidin-HRP immunoblot.** A-eggs; B- miracidia; C-daughter sporocyst; D-cercariae; E-lung stage somules; F-lung control; G-adult worms. An additional lung control lane (between lanes F and G) was cropped from the image.
(TIF)

Figure S3 **SmGAPDH is stably expressed throughout the** ***S. mansoni*** **life cycle.** Threshold values are plotted for SmGADPH amplification for each life cycle stage. Identical mRNA starting concentrations were used to generate template for each reaction. Two biological replicates are shown, and each was performed in triplicate. The first biological replicate was used as the reference gene for determining fold change of SmCE presented in Figure 1. The SmGAPDH primer/probe set non-specifically amplifies mouse GAPDH transcript, resulting in a Ct value for this sample.
(TIF)

Figure S4 **Anti-SmCE antibody and biotin-nVPL-(OPh)_2_ do not cross-react with uninfected snail tissue.** A. Ponceau stain of immunoblot and avidin-HRP membrane. B. Immunoblot (top) and avidin-HRP blot (bottom) of uninfected snail hepatopancreas (−) and 40 *d.p.i* snail hepatopancreas (+).
(TIF)

Figure S5 **Modeling of qualitative electrostatic potential of the surface of SmCE1a.1 and SmCE2b shows distinct differences in areas proximal to the active site.** A qualitative electrostatic representation of the 2 isoforms SmCE1a.2 (representative of the SmCE1 group) and SmCE2b (most different from rest of the isoforms) was generated using Pymol (The PyMOL Molecular Graphics System, Version 1.3, Schrödinger, LLC.). SmCE 1a.1 and SmCE2b were modeled on bovine chymotrypsin (PDB ID: 4CHA), and the known SmCE inhibitor, AAPF-CMK (yellow) is docked in the active site of the proteases.
(TIF)

Figure S6 **Protein alignment of all CE isoforms from** ***S. mansoni*** **,** ***S. haematobium*** **and** ***S. japonicum*** **.** The alignment was generated using ClustalW [17]. Protein sequences were obtained from schistoDB.net, NCBI Protein Database and Salter et al [7] .
(TIF)
