# Supplementary figures and images for: Investigation of the Proteolytic Functions of an Expanded Cercarial Elastase Gene Family in Schistosoma mansoni
Source: PLoS Negl Trop Dis. 2012 Apr 3;6(4):e1589. doi: 10.1371/journal.pntd.0001589 (PMC3317910; doi:10.1371/journal.pntd.0001589)

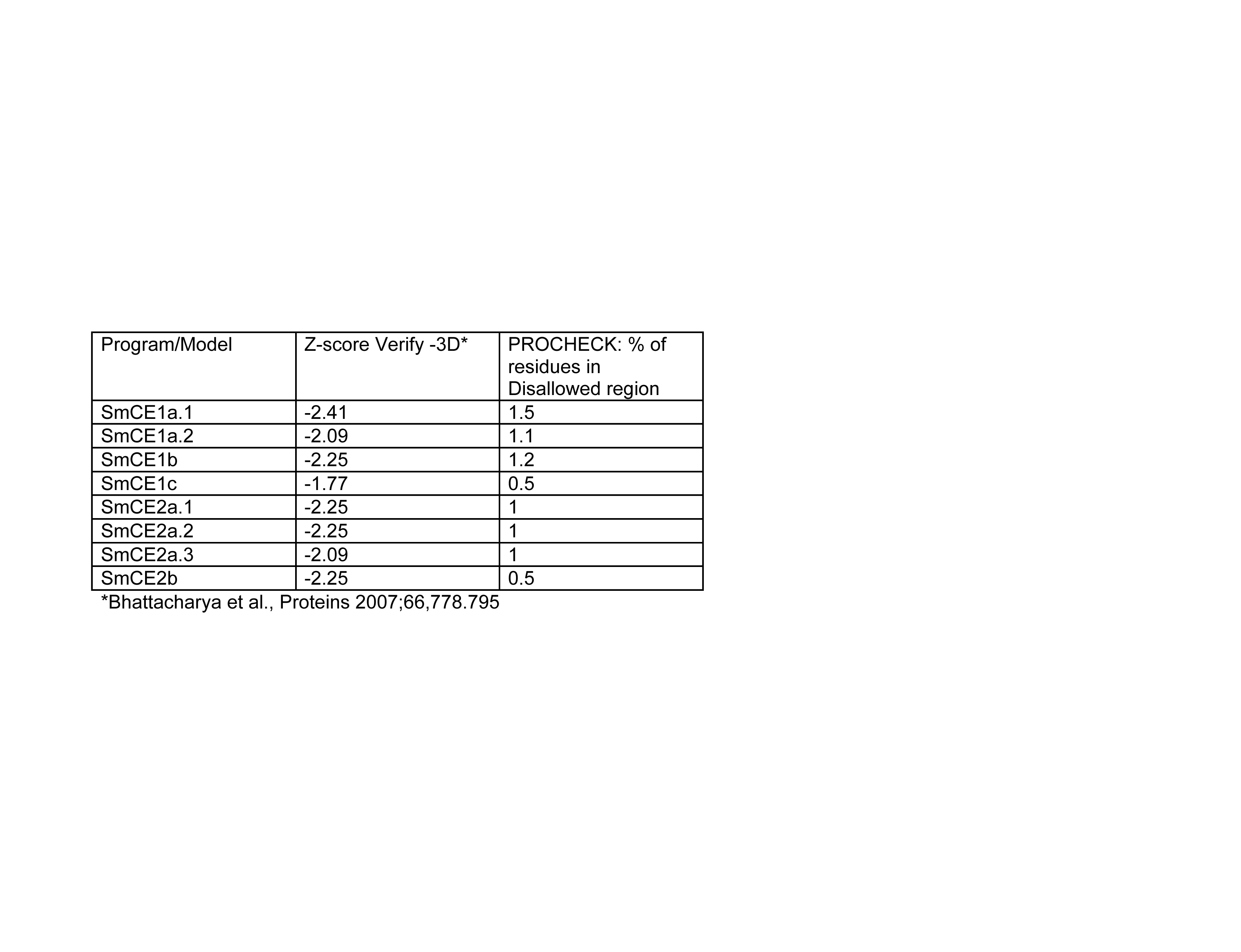

Supplement: Table S1 — Z-scores of models. The Protein Structure Validation Software Suite (PSVS server) was used to validate our models. There is no clear threshold score to define ‘good’ and ‘bad’ structures. However, since 2006 the NorthEast Structural Genomics Consortium has required that all NMR and X-ray crystal structures have Z scores>−5. (TIF) [file pntd.0001589.s001.tif]

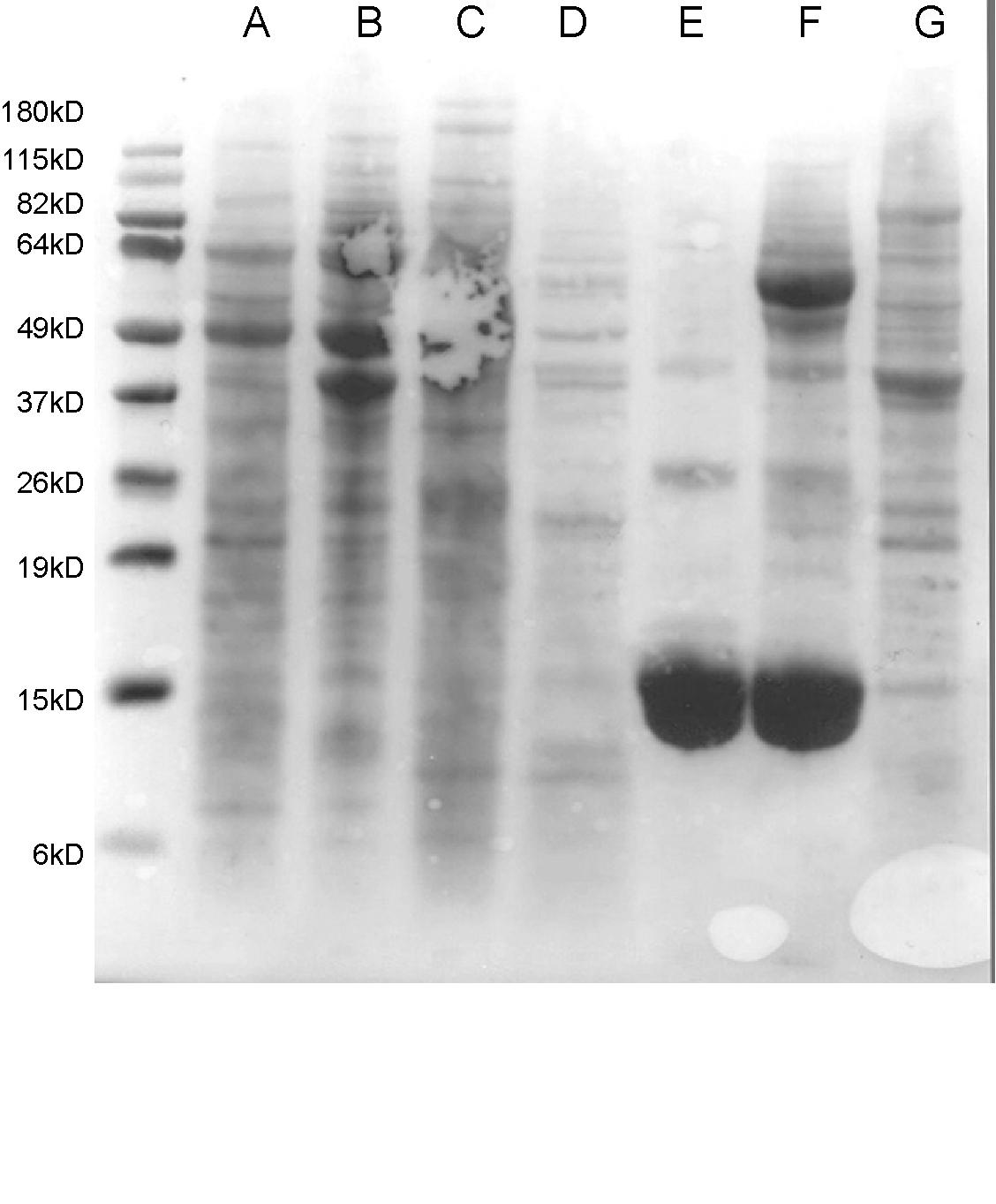

Supplement: Figure S1 — Ponceau stain of anti-SmCE immunoblot. A-eggs; B- miracidia; C-daughter sporocyst; D-cercariae; E-lung stage somules; F-lung control; G-adult worms. (TIF) [file pntd.0001589.s002.tif]

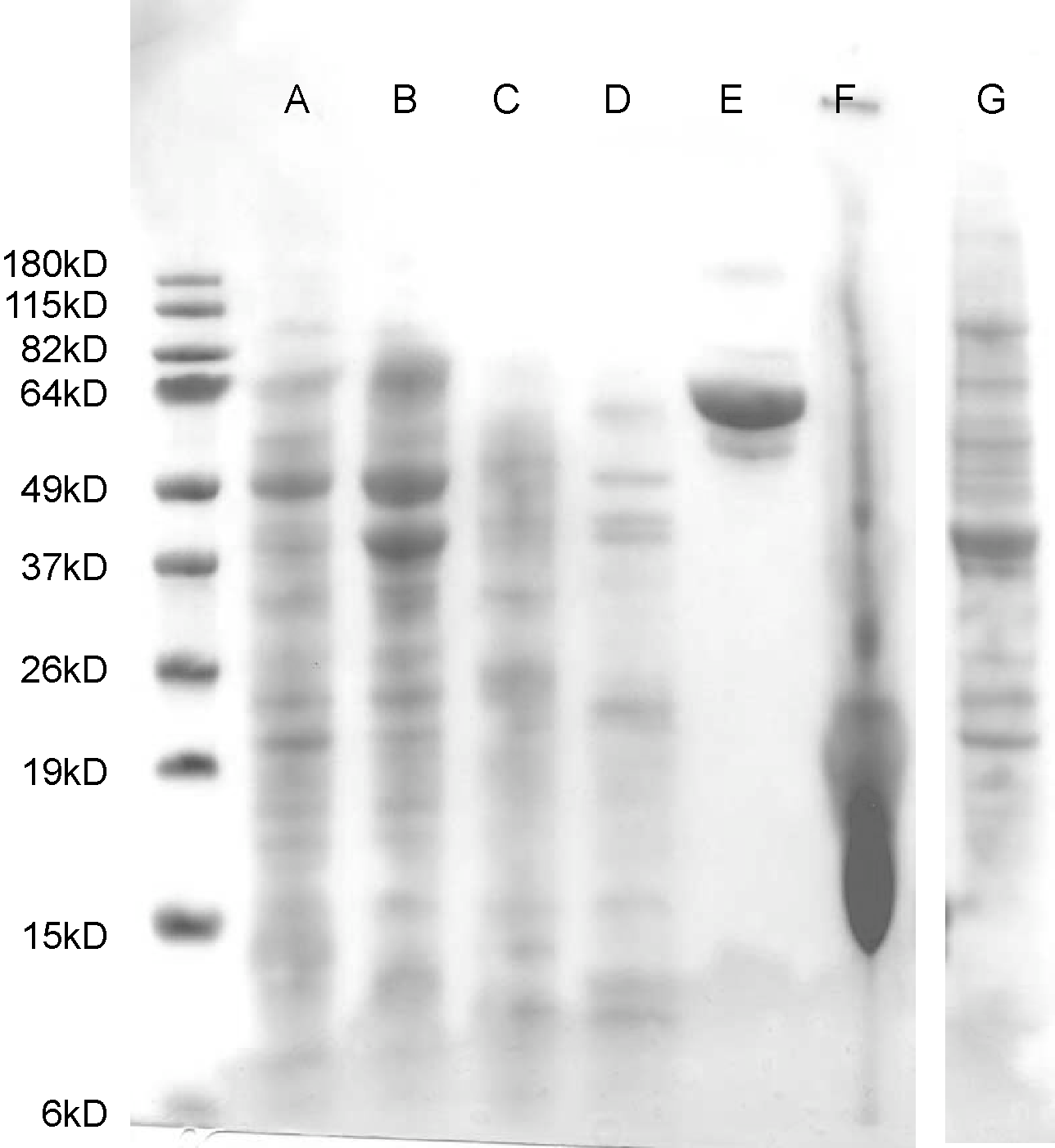

Supplement: Figure S2 — Ponceau stain of avidin-HRP immunoblot. A-eggs; B- miracidia; C-daughter sporocyst; D-cercariae; E-lung stage somules; F-lung control; G-adult worms. An additional lung control lane (between lanes F and G) was cropped from the image. (TIF) [file pntd.0001589.s003.tif]

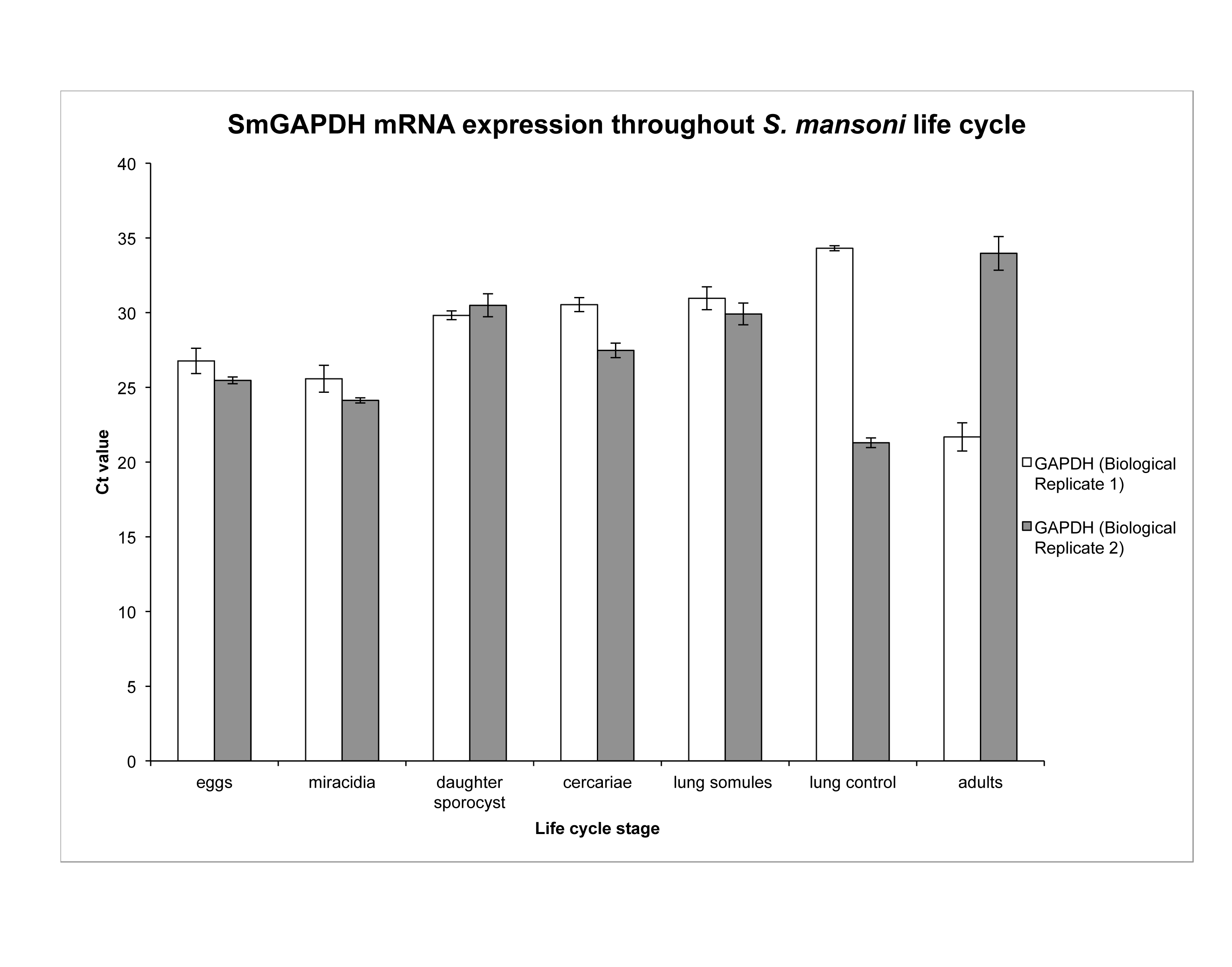

Supplement: Figure S3 — SmGAPDH is stably expressed throughout the S. mansoni life cycle. Threshold values are plotted for SmGADPH amplification for each life cycle stage. Identical mRNA starting concentrations were used to generate template for each reaction. Two biological replicates are shown, and each was performed in triplicate. The first biological replicate was used as the reference gene for determining fold change of SmCE presented in Figure 1. The SmGAPDH primer/probe set non-specifically amplifies mouse GAPDH transcript, resulting in a Ct value for this sample. (TIF) [file pntd.0001589.s004.tif]

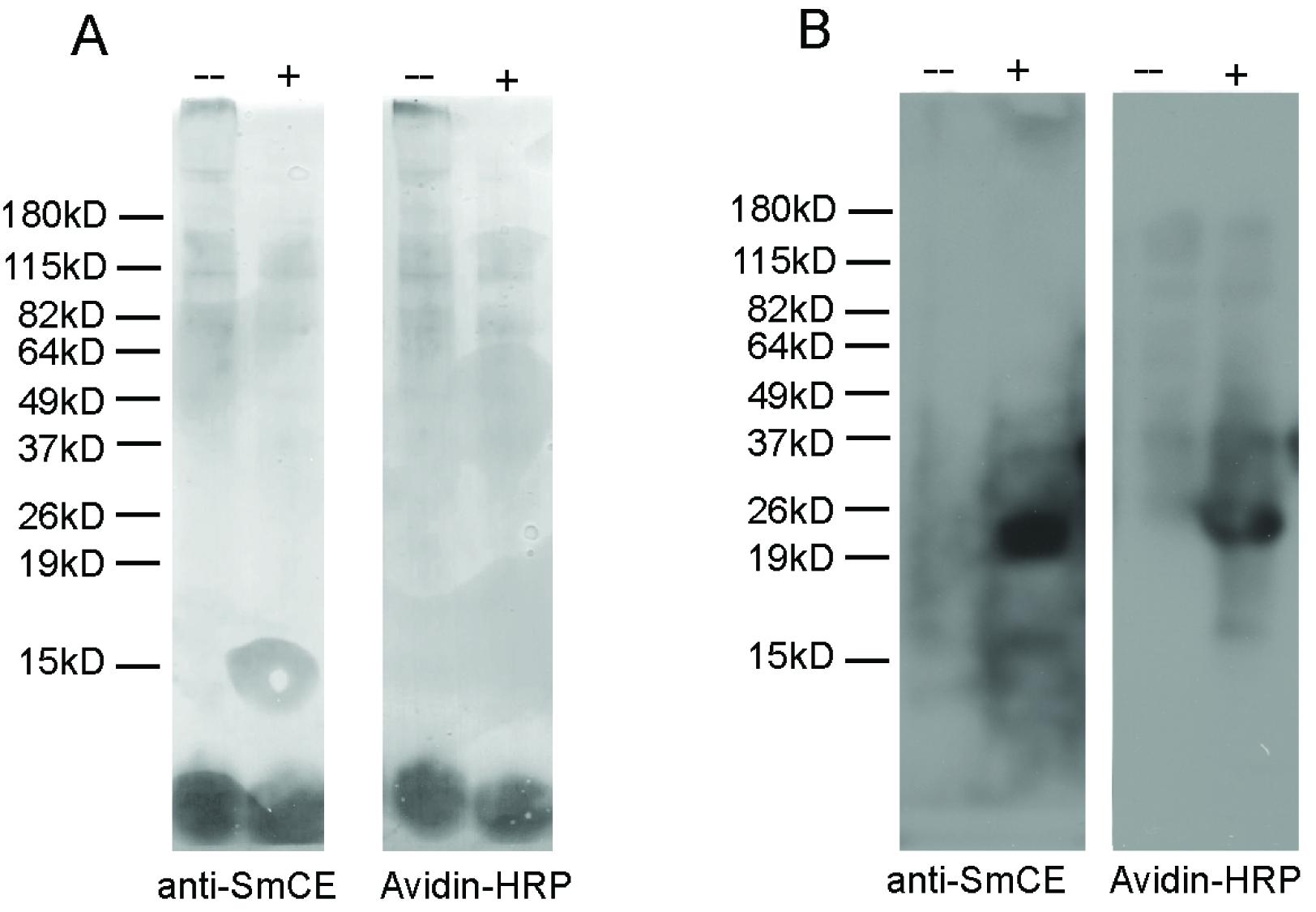

Supplement: Figure S4 — Anti-SmCE antibody and biotin-nVPL-(OPh)2 do not cross-react with uninfected snail tissue. A. Ponceau stain of immunoblot and avidin-HRP membrane. B. Immunoblot (top) and avidin-HRP blot (bottom) of uninfected snail hepatopancreas (−) and 40 d.p.i snail hepatopancreas (+). (TIF) [file pntd.0001589.s005.tif]

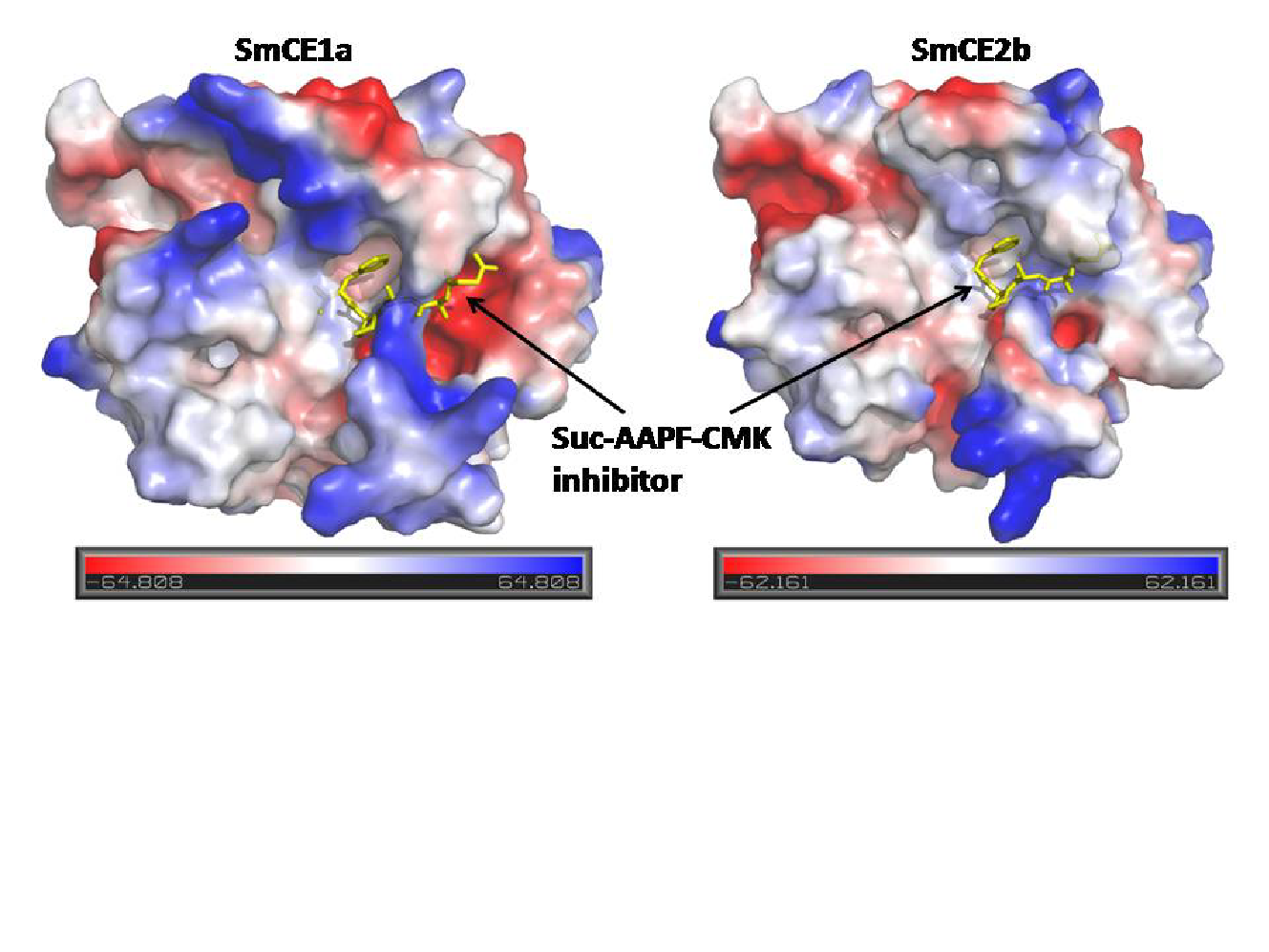

Supplement: Figure S5 — Modeling of qualitative electrostatic potential of the surface of SmCE1a.1 and SmCE2b shows distinct differences in areas proximal to the active site. A qualitative electrostatic representation of the 2 isoforms SmCE1a.2 (representative of the SmCE1 group) and SmCE2b (most different from rest of the isoforms) was generated using Pymol (The PyMOL Molecular Graphics System, Version 1.3, Schrödinger, LLC.). SmCE 1a.1 and SmCE2b were modeled on bovine chymotrypsin (PDB ID: 4CHA), and the known SmCE inhibitor, AAPF-CMK (yellow) is docked in the active site of the proteases. (TIF) [file pntd.0001589.s006.tif]

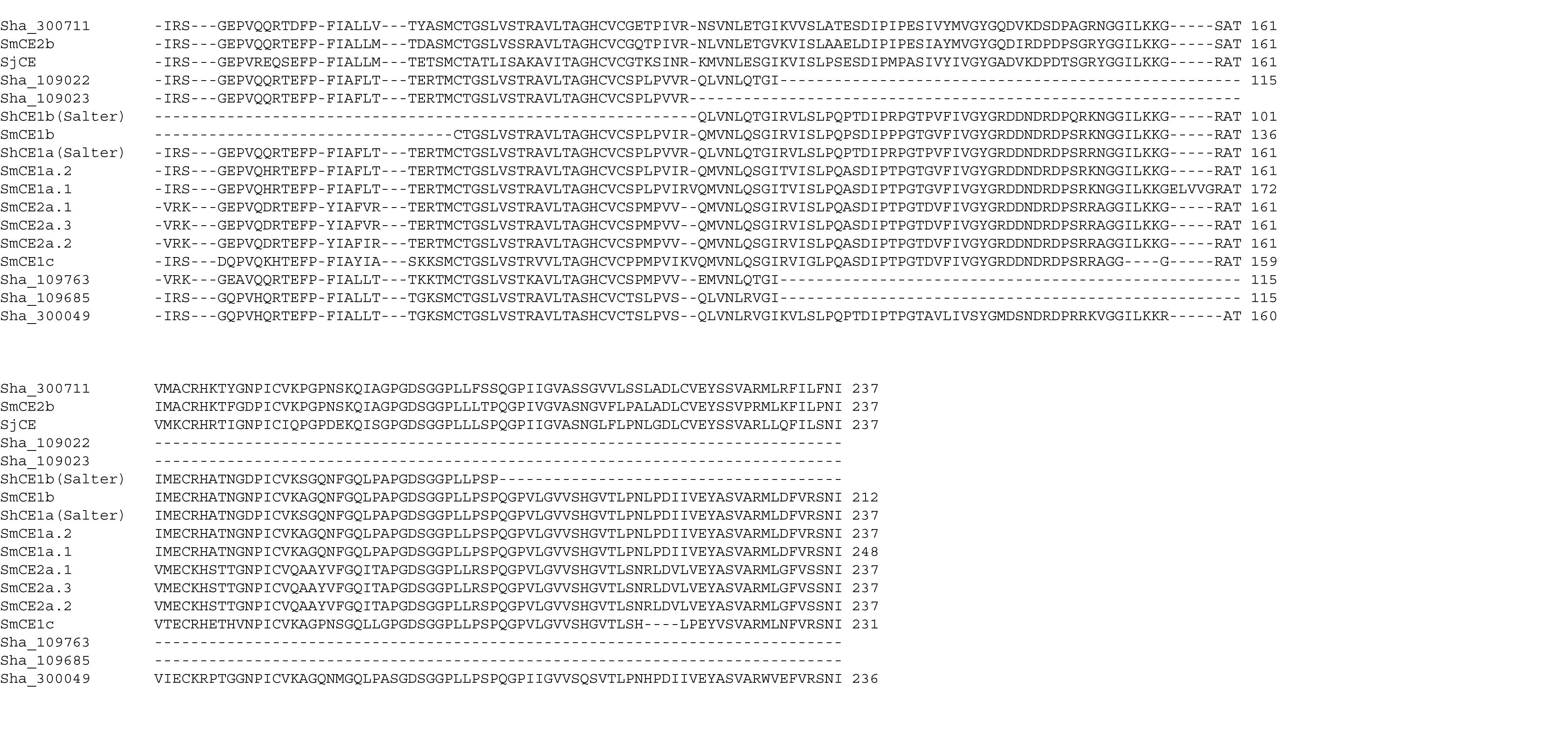

Supplement: Figure S6 — Protein alignment of all CE isoforms from S. mansoni , S. haematobium and S. japonicum . The alignment was generated using ClustalW [17]. Protein sequences were obtained from schistoDB.net, NCBI Protein Database and Salter et al [7] . (TIF) [file pntd.0001589.s007.tif]
